# Privacy Assessment in Mobile Health Apps: Scoping Review

**DOI:** 10.2196/18868

**Published:** 2020-07-02

**Authors:** Jaime Benjumea, Jorge Ropero, Octavio Rivera-Romero, Enrique Dorronzoro-Zubiete, Alejandro Carrasco

**Affiliations:** 1 Department of Electronic Technology Universidad de Sevilla Seville Spain

**Keywords:** privacy, mHealth, apps, privacy assessment, data privacy, review, security, mobile phone

## Abstract

**Background:**

Privacy has always been a concern, especially in the health domain. The proliferation of mobile health (mHealth) apps has led to a large amount of sensitive data being generated. Some authors have performed privacy assessments of mHealth apps. They have evaluated diverse privacy components; however, different authors have used different criteria for their assessments.

**Objective:**

This scoping review aims to understand how privacy is assessed for mHealth apps, focusing on the components, scales, criteria, and scoring methods used. A simple taxonomy to categorize the privacy assessments of mHealth apps based on component evaluation is also proposed.

**Methods:**

We followed the methodology defined by Arksey and O’Malley to conduct a scoping review. Included studies were categorized based on the privacy component, which was assessed using the proposed taxonomy.

**Results:**

The database searches retrieved a total of 710 citations—24 of them met the defined selection criteria, and data were extracted from them. Even though the inclusion criteria considered articles published since 2009, all the studies that were ultimately included were published from 2014 onward. Although 12 papers out of 24 (50%) analyzed only privacy, 8 (33%) analyzed both privacy and security. Moreover, 4 papers (17%) analyzed full apps, with privacy being just part of the assessment. The evaluation criteria used by authors were heterogeneous and were based on their experience, the literature, and/or existing legal frameworks. Regarding the set of items used for the assessments, each article defined a different one. Items included app permissions, analysis of the destination, analysis of the content of communications, study of the privacy policy, use of remote storage, and existence of a password to access the app, among many others. Most of the included studies provided a scoring method that enables the comparison of privacy among apps.

**Conclusions:**

The privacy assessment of mHealth apps is a complex task, as the criteria used by different authors for their evaluations are very heterogeneous. Although some studies about privacy assessment have been conducted, a very large set of items to evaluate privacy has been used up until now. In-app information and privacy policies are primarily utilized by the scientific community to extract privacy information from mHealth apps. The creation of a scale based on more objective criteria is a desirable step forward for privacy assessment in the future.

## Introduction

Although data privacy has always been a concern of the utmost interest, there has been some neglect for years, as changes have taken shape faster than regulations. Only recently have developers and customers really begun to worry about data privacy. The enormous amount of data handled by companies and the exposure of users’ sensitive information have led governments to design frameworks to care for the privacy of citizens [1,2]. Likewise, the large amount of data handled by the Internet of Things through big data techniques has raised concerns about privacy [3,4].

The health domain, however, was probably the first to have privacy regulation. In 1996, the Health Insurance Portability and Accountability Act (HIPAA) required the United States Department of Health and Human Services to safeguard protected health information according to national standards. Some of the requirements deal with data privacy [5].

In Europe, concerns have not been limited to the health domain, and regulations are strict. In 2018, the General Data Protection Regulation (GDPR) replaced the existing 1995 Data Protection Directive, and it became directly applicable to all European Union member states [6]. The GDPR introduced an important and modern change of approach toward a reinforced principle of accountability [7].

These concerns also apply to mobile health (mHealth) apps. mHealth technology has been widely adopted in many countries worldwide, as the number of smartphones and mHealth apps has increased dramatically. In 2018 in the United States, 77% of the population owned a smartphone [8], and in 2017, there were more than 300,000 mHealth apps [9]. The proliferation of this kind of app has allowed individuals to generate significant quantities of data about their lifestyles [10]. This situation has not escaped the attention of scientific researchers, and data privacy is a recurrent topic reported on in qualitative studies focused the needs and preferences of people with chronic conditions regarding mHealth solutions [11].

Although mHealth apps hold promise as self-management, monitoring, and behavior-change tools, among others, many smartphone users do not download mHealth apps because of lack of interest, cost, and concern about apps collecting their data [12]. Some studies have proven that there is cause for users’ concerns about both the privacy and security of these apps [13] and some assess only the lack of privacy of several of these apps [14,15]. It is, therefore, important to have the right tools to evaluate privacy and security levels by identifying different methods of assessing mHealth apps.

Despite privacy assessment currently being a relevant topic, there is a lack of objective protocols, methods, and procedures in place to define the necessary metrics and steps for a privacy assessment of an mHealth app. Different methods may be used to analyze privacy, such as assessment of privacy policies, evaluation of app communications, and studying app behavior. Extracting the information used to evaluate the privacy of mHealth apps, and even creating a taxonomy of the privacy components used for the assessment, should be important goals for researchers.

Further, different metrics and items have also been proposed to assess privacy. The types of measurements and items used should be based on laws, recommendations, and best practices. Discovering the different criteria that can be used for privacy assessment and the methods of defining them is imperative. Therefore, our literature review fills this research gap, focusing on describing and comparing how privacy is assessed by researchers in the mHealth domain.

Finally, we consider whether there is any measure of how good the privacy is in an mHealth app and how it would be possible to develop a scale for a privacy score. As such, we must search for any available way of assessing privacy in mHealth apps as well as the information that could potentially be used, and how it has been used, in these evaluations. To the best of our knowledge, no other review regarding the privacy of mHealth apps has been published.

## Methods

### Overview

This review aims to summarize how privacy is assessed in the literature including any type of study design. For this purpose, we conducted a scoping review using Arksey and O’Malley’s proposed framework [16]. We used Tricco et al’s PRISMA ScR (Preferred Reporting Items for Systematic Reviews and Meta-Analyses extension for Scoping Reviews) checklist [17] as a guide for reporting the procedure (see Multimedia Appendix 1). The authors of the framework include “summarize and disseminate research findings” and “identify research gaps in the existing literature” in the rationale for conducting a scoping review. Also, Arksey and O’Malley list “addressing a broad topic where many different study designs might be applicable” as a characteristic of scoping studies.

### Search Strategy

A systematic search strategy was used to identify relevant papers about the assessment of mHealth app privacy. The search was conducted in July 2019 in English, using terms regarding privacy, mHealth, and assessment; the following electronic databases were used: Scopus, PubMed, IEEE (Institute of Electrical and Electronics Engineers) Xplore, and ACM (Association for Computing Machinery) Digital Library. The search string used was as follows: privacy AND (“health app” OR “health apps” OR “mobile health” OR mhealth) AND (test OR testing OR tested OR framework OR review OR reviewing OR reviewed OR evaluate OR evaluation OR evaluating OR evaluated OR assess OR assessing OR assessment OR assessed OR “comparative analysis” OR “regulation compliance” OR taxonomy). The search terms and strategies for each database are detailed in Multimedia Appendix 2.

The database results were imported into the Mendeley application to further scrutinize the papers.

### Selection Criteria

The inclusion criteria for studies were as follows:

1. Papers that assessed the privacy of mHealth apps, regardless of the subject of the assessment, as well as papers that assessed several aspects of mHealth apps, including privacy.
2. Papers published with a title and abstract in English from 2009 onward in research journals, conference proceedings, or book chapters.

Papers that did not propose a method to evaluate privacy were excluded—even if they analyzed privacy—if they focused only on general aspects, such as users’ concerns, threat analysis, or challenges identified. Papers that did not evaluate any app were also excluded.

### Study Selection

After completing the search process and removing duplicates, the remaining 480 papers were screened. Initially, two authors (JB and JR) independently reviewed 10.0% (48/480) of the titles and abstracts to assess the level of agreement; the Cohen κ statistic, a measure of interrater reliability, was 0.73, which denotes an acceptable level of agreement [18]. Then, each author analyzed half of the remaining titles and abstracts to determine if they were potentially suitable for our objective. As a result, 77 articles were selected. Each author subsequently conducted a full-text review of those papers and 24 articles were ultimately included for data extraction. During this process, any doubt or discrepancy was resolved by consensus.

### Charting

The authors followed a collaborative and iterative process to define a charting table for collecting the data from the included studies. Information was gathered into four main groups: general information, evaluation procedure, evaluation criteria, and scoring method. Multimedia Appendix 3 shows the charting table that was used.

The *general information* group includes the year of publication, source title and type, app area, as well as the number of analyzed mHealth apps.

The *evaluation procedure* group comprises all the information related to the way the apps were assessed, according to the assessment design and the object of assessment. The assessment design deals with the type of evaluation that was done. Some papers analyze only privacy, while others assess security and privacy, and some even evaluate privacy as part of the whole functioning of the app. Information regarding whether the study assessed only app privacy, or whether app privacy was a component of a multidimensional evaluation, is included in this category. Additionally, information regarding what privacy components were assessed is also part of this group. After reviewing the full text of the included studies, a taxonomy of privacy components was defined by consensus. The categories, based on our review, that were used for the assessment of privacy were as follows:

1. App properties and behavior: this category refers to the app functionality. An article falls into this category if the app was actively used and some user information was provided to the app. Examples of this category are the type of log in used by the app, such as email or connecting via an external provider like Facebook, or if user registration and/or a password are needed to use the app.
2. In-app information: as with the previous category, the app was analyzed from within to look for information related to privacy, such as security measures or data sharing. Privacy policies were assessed in a separate item because some articles assess this in that fashion.
3. Personal information types: to fall into this category, the article must explicitly analyze the type of personal data collected by the app.
4. App communications: some articles analyze whether the app communications are private by intercepting traffic. Therefore, it is possible not only to know if traffic is encrypted but also, in some cases, to check the content of the traffic. Some authors were also able to find out the traffic destination of app communications, such as third parties and ad sites.
5. Static and dynamic analyses: the use of static and/or dynamic analysis is very common when evaluating the security of an app; however, these analyses can also be used to analyze certain aspects of privacy, such as whether privacy measures are properly implemented in app communications and the types of permissions used by an app.
6. Existence of a privacy policy: articles that check for the existence of a privacy policy are included in this category.
7. Analysis of the content of the privacy policy and/or the
  Type of Service: the authors of the article have read the privacy policy and searched for the presence or absence of certain information, such as how the data are stored, the use of encryption, and whether the data are shared with third parties, among others. Legibility (see the next category) is excluded from this category because the metrics used to evaluate legibility do not depend on the type of document being assessed.
8. Privacy policy legibility: transparency is one of the pillars of GDPR, and some articles analyze certain metrics regarding the readability of an app’s privacy policy, including the length of the document, number of phrases, and use of readability algorithms available in the literature.

The *evaluation criteria* group includes the items used for the assessment and what the assessment criteria are based on. Very heterogeneous information was extracted from each article, and the assessment criteria were decided on in varied ways. Evaluators chose a set of criteria based on the literature, the authors’ experience, an existing legal framework, and/or certain privacy recommendations and principles. It is difficult to categorize the criteria that were used to assess privacy, as they were not selected in a purely objective way. Different privacy items are defined according to the categories previously described. After extracting all the data regarding privacy assessment criteria from the studies that met the inclusion criteria, we defined, by consensus, a classification system consisting of 21 elements, listed hereafter.

A privacy policy is important when assessing privacy. The following items can be defined according to the content of a privacy policy: the existence of a data controller, details about the provision of a data protection officer, stating the purpose of data processing, establishing the legal basis, identifying the recipients of personal data, disclosing the occurrence of international data transfers, establishing the subject’s data rights (including the right to withdraw consent), whether it is an obligation to provide data, disclosing the occurrence of data profiling, detailing the nature of the collected information, stating the risks of data collection, disclosing the location of the collected information, and using anonymization.

Some of these items may also be defined by in-app information. Details regarding the purpose of data processing, the legal basis, the recipients of personal data, the existence of the subject’s data rights, the risks of data collection, and the protection of minors were extracted from the in-app information for this review.

Personal information types were used to define the nature and location of the collected information. App properties and behavior define whether user registration is necessary and the minimum amount of data collection that must be collected for an app to function correctly. App communications as well as static and dynamic analyses were used to check traffic and whether security measures were implemented; for these last cases, the distinction between security and privacy was not obvious.

Last, the *scoring method* group deals with the existence or nonexistence of a final score in each article. If there was a score, the weighting of assessed items was also considered.

The charting table containing all the data to be extracted was implemented using Microsoft Excel. Two authors (JB and JR) independently extracted data from the 24 selected articles. Discrepancies were resolved by consensus.

## Results

### Search Results

**Figure 1:**
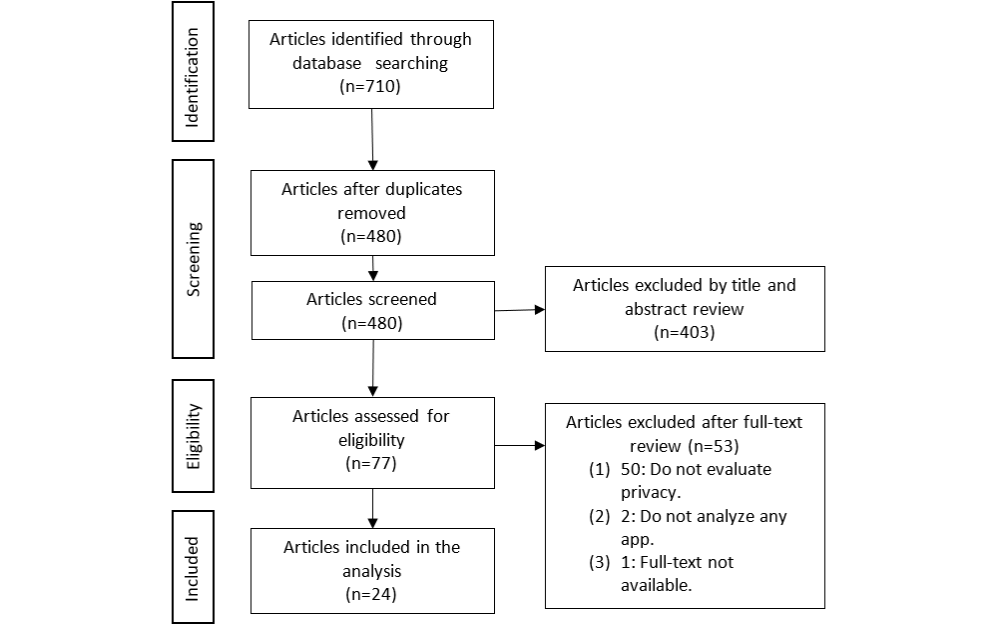
Flow diagram of the search strategy.

The database search retrieved a total of 710 citations; 230 duplicates were removed. After an initial screening of the abstracts and titles, 403 articles that did not meet the eligibility criteria were excluded and 77 were selected for full-text screening. After the full-text review, 24 studies [6,14,15,19-39] remained that fulfilled the inclusion criteria for this scoping review (see Figure 1). A full list of the included studies can be found in Multimedia Appendix 4.

### General Information

The general information contained in each study is summarized in Table 1. The year of publication, source title and type, app areas, and number of analyzed apps comprise the general information from each article. The source type is categorized as either a journal article, conference paper, or book chapter. The app areas were determined according to what the original article stated about the subject matter.

**Table 1:** General information from each article.

Reference	Source^a^	App areas	Number of analyzed apps
Papageorgiou et al, 2018 [6]	IEEE (Institute of Electrical and Electronics Engineers) Access (J)	Pregnancy and baby growth Family members and assistants Blood pressure and diabetes	20
Minen et al, 2018 [14]	Headache (J)	Headache	14
Huckvale et al, 2019 [15]	JAMA (Journal of the American Medical Association) Network Open (J)	Depression Smoking cessation	36
Scott et al, 2015 [19]	Australasian Journal of Information Systems (J)	General (top 20 mobile health [mHealth] apps)	20
Brüggemann et al, 2016 [20]	Annual Privacy Forum (J)	Medical Health and fitness	298
Mense et al, 2016 [21]	Studies in Health Technology and Informatics (BC)	Health and fitness	20
Hutton et al, 2018 [22]	JMIR mHealth and uHealth (J)	Self-tracking	64
Zapata et al, 2014 [23]	Annual International Conference of the IEEE Engineering in Medicine and Biology Society (C)	Personal health record	24
Sunyaev et al, 2015 [24]	Journal of the American Informatics Association (J)	Medical Health and fitness	600
Leigh et al, 2017 [25]	Evidence-Based Mental Health (J)	Chronic insomnia	19
Baumel et al, 2017 [26]	Journal of Medical Internet Research (J)	Health-related behaviors Mental health	84
Bachiri et al, 2018 [27]	Journal of Medical Systems (J)	Pregnancy	19
de las Aguas Robustillo Cortés et al, 2014 [28]	Telemedicine and e-Health (J)	HIV/AIDS	41
Quevedo-Rodríguez and Wagner, 2019 [29]	Endocrinología, Diabetes y Nutrición (J)	Diabetes	42
Knorr et al, 2015 [30]	IFIP (International Federation for Information Processing) Advances in Information and Communication Technology (J)	Diabetes Blood pressure	154
Zapata et al, 2014 [31]	RISTI (Revista Ibérica de Sistemas e Tecnologias de Informação) (J)	Personal health record	24
Bondaronek et al, 2018 [32]	JMIR mHealth and uHealth (J)	Physical activity	65
O’Laughlin et al, 2019 [33]	Internet Interventions (J)	Depression	116
Adhikari et al, 2014 [34]	Australasian Conference on Information Systems (C)	General (top 20 mHealth apps)	20
Aliasgari et al, 2018 [35]	IEEE Conference on Application, Information and Network Security (C)	General (top 25 mHealth apps)	25
Mense et al, 2016 [36]	Modeling and Simulation in Medicine Symposium (C)	Health and fitness	10
Powell el al, 2018 [37]	JMIR mHealth and uHealth (J)	Diabetes Mental health	70
Huckvale et al, 2015 [38]	BMC (BioMed Central) Medicine (J)	General	79
Robillard et al, 2019 [39]	Internet Interventions (J)	Mental health	369

^a^Sources include journal articles (J), conference papers (C), or book chapters (BC).

According to the type of source, 19 out of the 24 articles (79%) were published in journals [6,14,15,19,20,22,24-33,37-39], whereas 4 (17%) were published in conference proceedings [23,34-36] and 1 (4%) was a book chapter [21]. The publication fields were quite heterogeneous, with 12 out of 24 articles (50%) pertaining to the area of *medical informatics* [21-24,26-28,32,33,36,37,39], 5 (21%) to *medicine* [14,15,25,29,38], 4 (17%) to *information technology* [19,30,31,34], 2 (8%) to *security and privacy* [20,35], and 1 (4%) to a multidisciplinary source [6].

Based on our defined inclusion criteria, we analyzed articles published between January 2009 and July 2019. None of the selected articles was published between 2009 and 2013. Out of the 24 papers, 4 (17%) were published in 2014 [23,28,31,34], 4 (17%) in 2015 [19,24,30,38], 3 (13%) in 2016 [20,21,36], 2 (8%) in 2017 [25,26], 7 (29%) in 2018 [6,14,22,27,32,35,37], and 4 (17%) in the first half of 2019 [15,29,33,39].

A wide range of app types was analyzed in the included studies, and some articles analyzed apps in different areas. For instance, in Knorr et al [30], both diabetes and blood pressure apps were analyzed. Fitness apps, including self-tracking and physical activity apps, were the most analyzed, appearing in 6 articles (25%) [20-22,24,32,36]. Mental health apps, including apps for depression monitoring, were assessed in 5 articles (21%) [15,26,33,37,39], and diabetes-related apps appeared in 4 articles (17%) [6,29,30,37]. Other app areas were HIV/AIDS (1/24, 4%) [28], headache (1/24, 4%) [14], pregnancy and baby growth (2/24, 8%) [6,27], personal health record management (2/24, 8%) [23,31], chronic insomnia (1/24, 4%) [25], and smoking cessation (1/24, 4%) [15]. Top mHealth apps were assessed in 4 articles (17%) [19,34,35,38].

Only 2 articles out of 24 (8%) analyzed certified apps. Huckvale et al [38] analyzed 79 apps certified by the United Kingdom’s National Health Service (NHS) and concluded that there were gaps in compliance with data protection principles in these accredited apps. By contrast, Leigh et al analyzed 18 apps for Android and 1 NHS-certified app for iOS [25], and the authors found that the NHS-approved app outscored the others when using their evaluation criteria.

Finally, the number of apps analyzed in each article is disparate, ranging from 10 to 600 apps, with 20 apps being the mode (3/24, 13%) [19,34,35]. The average number of apps assessed was 92.6 (SD 136.9). Most of the articles (13/24, 54%), however, assessed less than 51 apps [6,14,15,19,21,25,27-29,31,34-36].

### Evaluation Procedure

A summary of the collected information is shown in Table 2. The objects of the assessments and the basis of the assessment criteria are described in the Methods section.

**Table 2:** Procedure for evaluation of the apps.

Reference	Area of assessment	Object of the assessment	Basis of the assessment criteria (includes legal framework)
Papageorgiou et al, 2018 [6]	Privacy and security	In-app information Static and dynamic analyses App communications Existence of a privacy policy Content of the privacy policy	Authors Legal
Minen et al, 2018 [14]	Privacy	Static and dynamic analyses App communications Existence of a privacy policy Content of the privacy policy	Authors
Huckvale et al, 2019 [15]	Privacy	In-app information App communications Existence of a privacy policy Content of the privacy policy	Literature
Scott et al, 2015 [19]	Privacy and security	App properties and behavior Existence of a privacy policy	Literature
Brüggemann et al, 2016 [20]	Privacy	App properties and behavior Personal information types App communications	Authors Literature
Mense et al, 2016 [21]	Privacy	App communications	Author
Hutton et al, 2018 [22]	Privacy	App properties and behavior In-app information Existence of a privacy policy Content of the privacy policy	Literature Legal Recommendations or principles
Zapata et al, 2014 [23]	Privacy	App properties and behavior Existence of a privacy policy	Literature Legal
Sunyaev et al, 2015 [24]	Privacy	Existence of a privacy policy Content of the privacy policy Legibility of the privacy policy	Authors
Leigh et al, 2017 [25]	Multidimensional	In-app information Existence of a privacy policy Content of the privacy policy	Legal Recommendations or principles
Baumel et al, 2017 [26]	Multidimensional	Existence of a privacy policy Content of the privacy policy	Literature
Bachiri et al, 2018 [27]	Privacy	App properties and behavior Existence of a privacy policy	Literature Legal Recommendations or principles
de las Aguas Robustillo Cortés et al, 2014 [28]	Multidimensional	App properties and behavior In-app information	Recommendations or principles
Quevedo-Rodríguez and Wagner, 2019 [29]	Multidimensional	App properties and behavior In-app information Existence of a privacy policy Content of the privacy policy	Recommendations or principles
Knorr et al, 2015 [30]	Privacy and security	Legibility of the privacy policy	Recommendations or principles
Zapata et al, 2014 [31]	Privacy	App properties and behavior Existence of a privacy policy Content of the privacy policy	Authors Recommendations or principles
Bondaronek et al, 2018 [32]	Privacy and security	Existence of a privacy policy Content of the privacy policy	Recommendations or principles
O’Laughlin et al, 2019 [33]	Privacy	Existence of a privacy policy Content of the privacy policy	Authors
Adhikari et al, 2014 [34]	Privacy and security	App properties and behavior In-app information Existence of a privacy policy	Literature
Aliasgari et al, 2018 [35]	Privacy and security	App communications	Legal
Mense et al, 2016 [36]	Privacy and security	App communications	Authors Recommendations or principles
Powell el al, 2018 [37]	Privacy	Existence of a privacy policy Legibility of the privacy policy	Authors
Huckvale et al, 2015 [38]	Privacy and security	App properties and behavior In-app information Static and dynamic analyses App communications Existence of a privacy policy Content of the privacy policy	Legal
Robillard et al, 2019 [39]	Privacy	Existence of a privacy policy Content of the privacy policy Legibility of the privacy policy	Authors Literature

Of the 24 articles assessed, 12 (50%) [14,15,20-24,27,31,33,37,39] evaluated only privacy; 8 (33%) evaluated security features, together with privacy [6,19,30,32,34-36,38]; and 4 (17%) conducted a multidimensional assessment [25,26,28,29], with privacy being only part of the evaluation.

When considering the object of the assessment, 19 out of the 24 articles (79%) [6,14,15,19,22-27,29-34,37-39] used the privacy policy as part of the assessment or solely evaluated the privacy policy. App properties and behavior were used for assessment by 10 articles (42%) [14,19,20,22,23,27-29,34,38], and 8 papers (33%) used in-app information [6,15,22,25,28,29,34,38] or app communications [6,15,20,21,30,35,36,38] for privacy evaluation. Finally, only 2 articles (8%) each used personal information types [20,38] and static and dynamic analyses [6,30].

The selected articles used different bases to define criteria to assess privacy of mobile apps. Most of the papers combined some sources to determine the items for assessment. Out of 24 papers, 10 (42%) [15,19,20,22,23,26,27,34,37,39] used the literature to determine the items, while 9 (38%) [6,14,20,21,24,33,36,37,39] were based on the authors’ criteria. Not many of the papers used legal frameworks or regulations—only 3 out of 24 papers (13%) [23,27,35] used the HIPAA and just 2 (8%) [6,22] explicitly mentioned the GDPR as a basis for determining the assessment criteria, although none of them checked the GDPR compliance. However, out of 24 articles, 2 (8%) [25,38] did use the previous European privacy regulation (ie, the 1995 Data Protection Directive). A total of 12 other privacy frameworks, recommendations from certification organizations and standard associations, and privacy principles were used. Multimedia Appendix 5 shows a further analysis regarding the object of the privacy assessment in mHealth apps.

### Evaluation Criteria

The evaluation criteria are heterogeneous, as were the methods for defining them. Though a very brief summary of the criteria is shown in Table 3, they are described in more detail in Multimedia Appendix 6. The classification items proposed by the different articles to be used for evaluating app privacy are shown in Table 4.

**Table 3:** Criteria for evaluation of the apps.

Reference	Criteria	Assessment of criteria
Papageorgiou et al, 2018 [6]	Privacy policy: consent, user rights (ie, withdraw and portability), data protection officer, data collection, purpose, and transfer Permission and static analysis Data transmission: https, SSL (Secure Sockets Layer), and secure transmission	Number of apps that meet the different criteria
Minen et al, 2018 [14]	Account functionality Data storage Privacy policy: type of information collected, data sharing, protection of minors, data access, and user rights	Number of apps that meet the different criteria
Huckvale et al, 2019 [15]	Privacy policy availability Uses of data, data transfer, and data collection Mechanisms for security, how long data will be retained, cookies, user rights (ie, opt-out, consequences of not providing data, deletion, editing, and complaints), and protection of minors Identity of data controller Adherence to privacy policy	Percentage of apps that meet the different criteria
Scott et al, 2015 [19]	User registration and authentication Data storing and sharing Enable users to update, correct, and delete their data Data privacy and security measures and existence of privacy policy	Items 1-3: risk score (1 point if there is a risk); Items 4-9: safety score (1 point if it is safe)
Brüggemann et al, 2016 [20]	Information-sharing targets (S), information transfer (T), and information collection (U) Personal information types (P) and log-in (L) Connection security (R)	PrivacyRiskScoreApp = TApp × w(T) + PApp × w(P) + LApp × w(L)+ SApp × w(S) + UApp × w(U) + RApp × w(R) w = weight
Mense et al, 2016 [21]	Use of SSL and certificate pinning Information sent and identification of third parties	Number of apps that meet the different criteria
Hutton et al, 2018 [22]	Notice and awareness: data sharing, nature of data, and explanation of security measures Choice or consent: user-consent control Access or participation: user access to data Social disclosure: privacy control	Most heuristics are valued as 0-2 (0, 1, or 2), though some have slightly different values (ie, 0/1, 0-3, or 0-4)
Zapata et al, 2014 [23]	Privacy policy access and updates Authentication, encryption, and security standards Access can be granted and revoked	All six items are valued as 0, 0.5, or 1
Sunyaev et al, 2015 [24]	Privacy policy availability Privacy policy features: length, readability, scope, and transparency (ie, sharing, collection, and user controls)	Number of apps that meet the different criteria
Leigh et al, 2017 [25]	Data sharing Confidentiality mechanisms Privacy policy availability and content (ie, data collection, use of data, and data encryption)	App privacy features (1-2) and privacy policy (3-8), with 1 point per question
Baumel et al, 2017 [26]	Data communications, storage, and sharing Notification of how personal information is kept confidential Protection of minors Anonymization	Eight items: 1 point if the app does not include the item
Bachiri et al, 2018 [27]	Privacy policy location and updates Access management: permissions, audit, criteria, and authentication Security measures Consideration of the Health Insurance Portability and Accountability Act (HIPAA)	Number of criteria that are met (35 items)
de las Aguas Robustillo Cortés et al, 2014 [28]	Data transmission and confidentiality Registration, purpose of use, information disclosure, and social disclosure Protection of minors and mechanisms to avoid unauthorized access Information storage	–1 (does not meet the criterion), 0 (not applicable), or 1 (meets the criterion)
Quevedo-Rodríguez and Wagner, 2019 [29]	Nature and purpose of the information and data storing Information about privacy, consent, and security measures User access Protection of minors	Compliance with items: 2 (complies), 1­ (partially complies), or 0 (does not comply)
Knorr et al, 2015 [30]	Static and dynamic analyses and web connection Inspection of privacy policies	General compliance with the items
Zapata et al, 2014 [31]	Notification: privacy policy access and updates, cookies, and use of safety standards Security: authentication, encryption, server protection, and backup copies Election and access: access can be granted and revoked and access in case of emergency	Compliance with items: 2 (complies), 1­ (partially complies), or 0 (does not comply)
Bondaronek et al, 2018 [32]	Privacy information: availability, accessibility, data collecting, data sharing, and data security	Number of apps that meet the different criteria
O’Laughlin et al, 2019 [33]	Privacy policy availability, existence of a log-in process, and identification Data storage and sharing User access: editing and deletion	Some of the items received a white, light-grey, or dark-grey score; other items received a white or light-grey score; 1 item received a white, light-grey, or black score
Adhikari et al, 2014 [34]	User registration and authentication Data storing and sharing Enable users to update, correct, and delete their data Data privacy and security measures and existence of privacy policy	Items 1-3: risk score (1 point if there is a risk); Items 4-8: safety score (1 point if it is safe)
Aliasgari et al, 2018 [35]	SSL configuration Data transfer and collection Compliance with the HIPAA	HIPAA compliance or not: the authors checked if the terms and conditions indicated HIPAA compliance, or they asked the app’s support team
Mense et al, 2016 [36]	Encryption Data transmission	Number of apps that meet the different criteria
Powell el al, 2018 [37]	Privacy policy readability: word count, sentences per paragraph, words per sentence, characters per word, average number of sentences per 100 words, average words with 6 or more characters, Flesch Reading Ease, Flesch-Kincaid Grade Level, Gunning Fog Score, SMOG (Simple Measure of Gobbledygook) Index, Coleman Liau Index, Automated Readability Index, Fry Grade Level, and Raygor Estimate Graph Grade Level	Average score, median, or range for every item comparing diabetes apps vs mental health apps
Huckvale et al, 2015 [38]	Privacy policy: availability and features Concordance of privacy policies and data-handling practices Coverage of privacy policy: data collection, data transfer, anonymization, how long data are retained, use of cookies, user rights (ie, opt-out, consequences of not providing data, data access, and complaints), identification of data controller, and updates	Percentage of apps that meet the different criteria
Robillard et al, 2019 [39]	Collected information (ie, nature and types), use of information, and data sharing Reasons for disclosing information User rights: consent, opt-out, and deletion	Percentage of apps that meet the different criteria

**Table 4:** Items present in the assessment of criteria for each article.

Item	Reference																							
	[6]	[14]	[15]	[19]	[20]	[21]	[22]	[23]	[24]	[25]	[26]	[27]	[28]	[29]	[30]	[31]	[32]	[33]	[34]	[35]	[36]	[37]	[38]	[39]
Existence of a data controller	X		X				X																X	
DPO^a^ details are given	X																							
Purposes of the processing are stated		X	X				X			X	X		X	X	X		X	X			X		X	X
Legal basis exists	X		X				X	X		X		X		X		X							X	X
Recipients of personal data are identified		X	X	X		X	X		X	X			X	X	X		X	X	X		X		X	X
International data transfers are disclosed	X					X																		
Data storage period is stated			X																				X	
Existence of users’ data rights		X	X	X			X	X	X			X	X	X		X		X	X					X
Existence of the right to withdraw consent	X		X				X																	X
Existence of the right to complain to a supervisory authority			X																				X	
Obligation to provide data			X																				X	
Existence of data processing and profiling														X										
Nature of the collected information is disclosed		X			X	X	X				X		X	X										X
Risks of data collection and management of confidentiality breaches are stated										X	X													
Location of the collected information is disclosed		X		X	X		X												X					
User registration is required				X	X								X					X	X					
Existence of a privacy policy	X	X	X	X					X	X	X	X			X		X	X	X				X	
Privacy policy good practices	X		X					X	X			X			X	X	X					X	X	
Minimum data needed for app functioning are collected										X														
Protection of minors and age of verification exists		X	X								X		X	X										
Anonymization takes place		X	X								X	X					X							X

^a^DPO: data protection officer.

As seen in Table 4, many different items were considered as criteria to assess privacy. We have defined 21 items, but only four of them were taken into account by more than half the selected articles. The identification of the recipients of personal data was used as an evaluation criterion in 16 out of the 24 papers (67%) [14,15,19,21,22,24,25,28-30,32-34,36,38,39]. The existence of a privacy policy was determined by 13 out of 24 articles (54%) [6,14,15,19,24-27,30,32-34,38]. The stating of the purposes of the data processing was also examined by 13 papers (54%) [14,15,22,25,26,28-30,32,33,36,38,39]. Additionally, 13 articles (54%) [14,15,19,22-24,27-29,31,33,35,39] determined the existence of subjects’ data rights, though only partially—most of them only considered access and/or data control by the user.

Table 4 also shows two different ways of assessing privacy. Out of 24 papers, 10 (42%) [6,14,15,21,24,30,32,36,38,39] checked whether the analyzed apps met the criteria described in the Evaluation Procedure section. Meanwhile, 14 articles out of 24 (58%) [19,20,22,23,25-29,31,33-35,37] evaluated the different apps according to several criteria.

### Scoring Method

Of the 14 articles that assessed apps according to several items, 13 (93%) of them provided a scoring method that enables a comparison of privacy among apps. Only 1 paper (7%) [22] did not give a final score, although every item had an associated score; thus, a scoring method could easily be developed. The items were assessed in a binary manner in 6 out of the 14 papers (43%) [19,25-27,34,35], which produced a score. Out of 14 articles, 7 (50%) [14,20,22,23,28,29,33] used a binary assessment with intermediate values: 0, 0.5, or 1; 0, 1, or 2; or –1, 0, or 1 were used. Hutton et al utilized different discrete values depending on the assessed items [22]. Bondaronek et al used discrete values—white, light grey, dark grey, and black—to obtain a final score of acceptable, unacceptable, or questionable [32].

Focusing on the articles that developed a scoring method, we have also analyzed whether the scoring was weighted. In that case, all the items would have different weights according to their importance when calculating the final score. Only 2 articles out of 24 (8%) [20,28] proposed a weighted score and 1 article (4%) [6] distinguished between “major issues” and “minor issues” but did not produce a final score. A summary is shown in Table 5.

**Table 5:** Scoring methods used to assess apps.

Reference	Score	Weighted score
Papageorgiou et al, 2018 [6]	No	No, though there are “major issues” and “minor issues”
Minen et al, 2018 [14]	No	N/A^a^
Huckvale et al, 2019 [15]	No	N/A
Scott et al, 2015 [19]	Yes. Risk score: 0-3; safety score: 0-6	No
Brüggemann et al, 2016 [20]	Yes. Connection security (S), information-sharing targets (T), unspecific information transfer (U), information collection (R), and log-in (L) are binary. Personal information type (P) is more elaborated: 13 types are considered and a correction factor is applied.	Yes, it can be configured by the user
Mense et al, 2016 [21]	No	N/A
Hutton et al, 2018 [22]	The paper does not give a score but, rather, explains how different heuristics are implemented. However, it is easy to assign a score to every app with the available information.	N/A, although it can be calculated (see Scoring Method section above)
Zapata et al, 2014 [23]	Yes: 0-6	No
Sunyaev et al, 2015 [24]	No	N/A
Leigh et al, 2017 [25]	Yes: 0-8	No
Baumel et al, 2017 [26]	Yes: 0-8, with 0 points being maximum privacy	No
Bachiri et al, 2018 [27]	Yes: 0-35	No
de las Aguas Robustillo Cortés et al, 2014 [28]	Yes, but it is a general app score, not only for privacy	Yes, weighted by experts
Quevedo-Rodríguez and Wagner, 2019 [29]	Yes, but as part of the global app quality	No
Knorr et al, 2015 [30]	No	N/A
Zapata et al, 2014 [31]	Yes	No
Bondaronek et al, 2018 [32]	No, at least for the privacy items	N/A
O’Laughlin et al, 2019 [33]	Yes: acceptable, unacceptable, or questionable	No
Adhikari et al, 2014 [34]	Yes. Risk score: 0-3; safety score: 0-5	No
Aliasgari et al, 2018 [35]	Yes. Although there is no global score, there are certain scores pertaining to Transport Layer Security (TLS) and Health Insurance Portability and Accountability Act (HIPAA) compliance.	No
Mense et al, 2016 [36]	No	N/A
Powell el al, 2018 [37]	Average score, median, and range for every item	No
Huckvale et al, 2015 [38]	No	N/A
Robillard et al, 2019 [39]	No	N/A

^a^N/A: not applicable.

## Discussion

### General Information

This review deals with the privacy assessment for mHealth apps. Finding information about the assessment of privacy of mHealth apps is not a trivial task, as the sources are very heterogeneous, including many areas of application. What is obvious is that the interest in privacy has been growing in the scientific community, with special significance in recent years. Despite studying the period from 2009 to 2019, the 24 selected articles were published in 2014 or later.

Privacy is essential in the health domain, and the app areas are very diverse. Fitness, mental health, and diabetes apps were common in the assessments, but such varied fields as HIV/AIDS, pregnancy, and headaches were considered. Some papers, such as Powell et al, evaluated seemingly unrelated areas, such as mental health and diabetes, at the same time [37]. The number of analyzed apps per paper also varied widely, from 10 apps [36] to 600 apps [24].

### Evaluation Procedure

The articles presented in this scoping review evaluated privacy in different ways. Some of them analyzed only privacy, whereas others evaluated it together with security or other app functions.

Several of the articles used the privacy policy to determine information about the app privacy, but researchers should report more detailed information regarding how they assess the privacy of apps to ensure the reliability of their studies. As an example, it is not clear how so much information was obtained by analyzing only the app privacy policies in 3 papers (13%) [27,31,33]—perhaps an in-app information assessment was also performed. None of the articles explained how they evaluated privacy policies when considering certain items, such as informing the user about the secondary uses of their data. Some authors even noted that there were difficulties in evaluating privacy policies due to the complexity of the language used in them (eg, “Disagreements between the raters arose primarily from confusion over the apps’ privacy policies, which were often unclear in terms of language and intent” [22]), but none of them specified the exact criteria used to evaluate the content of the privacy policies. This could lead to inconsistent results if their assessment framework were to be used by others. Specifying the particular criteria used in the assessment could make the evaluations reproducible.

The legal framework is another important issue with privacy assessment. The number of mHealth apps has increased considerably [8], and important privacy regulations have emerged—not only in the mHealth domain—such as the GDPR [6,7]. However, only 7 out of the 24 articles (29%) used law as a direct source for establishing the assessment criteria—4 of them [6,22,25,38] used the European legislation (ie, the GDPR or the 1995 Data Protection Directive) as a source and 3 [23,27,35] were based on the HIPAA. Although some authors were skeptical about the applicability of the HIPAA to mHealth apps [6,40], others suggested that the HIPAA might be applicable [35]. If articles that used recommendations directly from private and/or public bodies, such as the US Federal Trade Commission or the UK Information Commissioner’s Office, are considered in this category, then the number of articles that contemplated laws goes up to 11 (46%). Additionally, data minimization is one of the main principles regarding processing personal data in the GDPR, meaning that data collection should be limited to processing purposes only. However, only 2 papers (8%) [20,38] analyzed the types of data collected by an app.

Several articles in our review also analyzed whether communications were secured, and 8 articles (33%) [6,15,20,21,30,35,36,38] actually checked if they were. Moreover, 1 article (4%) [15] brought to our attention that discrepancies between what the privacy policy states about app data transmission and the real data transmissions are not uncommon. By contrast, Huckvale et al did not observe any discrepancy [38]. Nevertheless, future analyses of privacy policies could verify whether developers properly disclose the nature of app communications.

Although the privacy policy is a common source of data to assess the privacy of apps, there are many challenges to address. The evaluation procedure needs to be straightforward by removing subjective and unclear assessments of privacy. It should also be supported by a legal framework, although that is not the current trend.

### Evaluation Criteria

The criteria that have been used to assess the privacy of mHealth apps are very diverse. We have identified 21 items but, within each item, there are particularities that depend on the authors’ criteria. Moreover, as previously mentioned, in many cases, the criteria used to assess the items are not explained clearly enough, or they are not easily reproducible. Therefore, the list of different items and how they are evaluated never ends, and it is extremely subjective. Although the evaluations in this review are useful, we suggest a more objective privacy assessment.

As an example, some articles searched for specific information in the privacy policy, such as whether the user is informed about other uses of their data, whereas other papers looked for this information in the app. We consider that it is possible to miss important information by searching in the wrong place. For instance, 2 articles (8%) [22,38] checked both elements—the privacy policy and the app—while 6 papers (25%) [14,24,28,30,32,33] only checked the app, with no reference to the privacy policy. In 4 papers (17%) [25,26,29,34] it was not clear whether it was the app or the privacy policy that was examined. Finally, 3 papers (13%) [6,15,39] used only the app privacy policy and the terms and conditions.

One of the main issues created by the subjectivity of the evaluation criteria involves the nature of the items used. Sometimes the criteria are not clear enough. This issue may lead to different results when other users and/or developers assess privacy. New evaluation approaches should put special emphasis on defining clear and objective items to evaluate.

### Scoring Method

A scoring method or scale to assess app privacy could be a key tool for systematically comparing apps. Many scoring methods were used in the included studies. Most of them are quite simple, with a methodology that consists of assigning a binary value to some defined items, but they have, nonetheless, proven to be effective in assessing privacy by providing a simple approach to comparing apps. A weighted score, which highlights the importance of some items over others, was also explored in 2 papers (8%).

Despite the promising results derived from the use of a weighted score, further research must be conducted to identify the subjective relevance and importance of the different items perceived by consumers, patients, and experts, in order to assess the privacy of the apps. Further research must also be conducted aimed at defining common legal-based criteria to better assess the privacy of mHealth apps.

### Review Limitations

This study has several limitations. Relevant studies may have been missed if they were published with a title or abstract in a language other than English, outside of the specified time frame, or in different databases than those that were used. Some studies may not be included due to the keywords chosen for the search string.

Specifically, for this review, the absence of an existing taxonomy of the privacy components used for the assessment is also a limitation. Although we attempt to compensate for this limitation with our level of expertise and detailed knowledge, charting is still subjective.

Finally, the different requirements implied by different types of apps shows that not all apps are equally sensitive to privacy risks, which suggests the possibility of analyzing how crucial privacy is according to the type of app. As we did not find any such existing classification system, we set this as a point for future research.

### Conclusions

Privacy in mHealth apps has been determined based on an analysis of the app user interface, communications privacy, and privacy policy. Checking privacy in communications is usually very straightforward, with objective criteria for its assessment. When analyzing user interfaces and privacy policies, however, the criteria are very heterogeneous and less objective; this is especially true when analyzing privacy policies, which can lead to irreproducible results. In our opinion, it is very important to develop a more detailed assessment of privacy policies, so that the assessment frameworks may be utilized by subsequent users and lead to coherent results.

Another important conclusion from this study is that there is a lack of analyses pertaining to the types of personal information collected by the apps. Minimization is one of the principles of the GDPR, so a greater effort should be made to analyze whether apps gather more personal information than is necessary.

In short, despite great progress made through the scientific community’s awareness about the importance of privacy assessment of mHealth apps, there is still a long way to go. A positive step forward would be the creation of a scale or scoring system based on objective criteria, which would, therefore, be less open to interpretation. Another good development would be the use of a certain legal basis for such a scale and explaining in detail how to apply the evaluation criteria.

## Appendix

Multimedia Appendix 1 PRISMA ScR (Preferred Reporting Items for Systematic Reviews and Meta-Analyses extension for Scoping Reviews).

Multimedia Appendix 2 Search terms and strategies for each database.

Multimedia Appendix 3 Charting table used for collecting data from the included studies.

Multimedia Appendix 4 Included studies.

Multimedia Appendix 5 Objects of assessment of the apps.

Multimedia Appendix 6 App data extraction details.

## Abbreviations

ACM: Association for Computing Machinery
GDPR: General Data Protection Regulation
HIPAA: Health Insurance Portability and Accountability Act
IEEE: Institute of Electrical and Electronics Engineers
mHealth: mobile health
NHS: National Health Service
PRISMA ScR: Preferred Reporting Items for Systematic Reviews and Meta-Analyses extension for Scoping Reviews
