# Supplementary material for: Privacy Assessment in Mobile Health Apps: Scoping Review
Source: JMIR Mhealth Uhealth. 2020 Jul 2;8(7):e18868. doi: 10.2196/18868 (PMC7367524; doi:10.2196/18868)
Supplement: Multimedia Appendix 2 [file mhealth_v8i7e18868_app2.docx]

| **Database** | **Search strategy** |
| --- | --- |
| Scopus | TITLE-ABS-KEY ( privacy ) AND TITLE-ABS-KEY ( "health apps" OR "health app" OR "mobile health" OR mhealth ) AND TITLE-ABS-KEY ( test OR testing OR tested OR framework OR review OR reviewing OR reviewed OR evaluate OR evaluation OR evaluating OR evaluated OR assess OR assessing OR assessment OR assessed OR "comparative analysis" OR "Regulation compliance" OR taxonomy ) |
| Pubmed | (privacy[Title/Abstract]) AND ("health app"[Title/Abstract] OR "health apps"[Title/Abstract] OR "Mobile health"[Title/Abstract] OR Mhealth[Title/Abstract]) AND (Test[Title/Abstract] OR testing[Title/Abstract] OR tested[Title/Abstract] OR Framework[Title/Abstract] OR Review[Title/Abstract] OR reviewing[Title/Abstract] OR reviewed[Title/Abstract] OR evaluate[Title/Abstract] OR evaluation[Title/Abstract] OR evaluating[Title/Abstract] OR evaluated[Title/Abstract] OR assess[Title/Abstract] OR assessing[Title/Abstract] OR assessment[Title/Abstract] OR assessed[Title/Abstract] OR "comparative analysis"[Title/Abstract] OR "Regulation compliance"[Title/Abstract] OR taxonomy[Title/Abstract]) |
| IEEE Xplore | privacy AND ("health app" OR "health apps" OR "mobile health" OR mhealth) AND (Test OR testing OR tested OR Framework OR Review OR reviewing OR reviewed OR evaluate OR evaluation OR evaluating OR evaluated OR assess OR assessing OR assessment OR assessed OR "comparative analysis" OR "Regulation compliance" OR taxonomy) |
| ACM-DL | acmdltitle: ( +(privacy) +("health apps" "health app" mhealth "mobile health") +(Test testing tested Framework Review reviewing reviewed evaluate evaluation evaluating evaluated assess assessing assessment assessed "comparative analysis" "Regulation compliance" taxonomy)) OR recordAbstract: ( +(privacy) +("health app" "health apps" mhealth "mobile health") +(Test testing tested Framework Review reviewing reviewed evaluate evaluation evaluating evaluated assess assessing assessment assessed "comparative analysis" "Regulation compliance" taxonomy)) OR keywords.author.keyword: ( +(privacy) +("health app" "health apps" mhealth "mobile health") +(Test testing tested Framework Review reviewing reviewed evaluate evaluation evaluating evaluated assess assessing assessment assessed "comparative analysis" "Regulation compliance" taxonomy)) |
