# Supplementary material for: Privacy Assessment in Mobile Health Apps: Scoping Review
Source: JMIR Mhealth Uhealth. 2020 Jul 2;8(7):e18868. doi: 10.2196/18868 (PMC7367524; doi:10.2196/18868)
Supplement: Multimedia Appendix 3 [file mhealth_v8i7e18868_app3.docx]

Charting Template

| **General Information** |
| --- |
| **Authors:** Reference to authors using citation style |
| **Title:** Title of the study |
| **Year:** Year of publication |
| **Source Title:** Journal or Conference Title |
| **Source Type:** Journal or Conference |
| **App area:** Areas (as authors defined) to which assessed apps belong |
| **Analyzed apps:** Number of apps assessed in the study |
| **Evaluation Procedure** |
| **Assessment Design**: Privacy (only), Privacy and security, or Multidimensional evaluation |
| **Object of assessment:** Privacy Components: (Select appropriate categories)   - **App properties and behavior:** This category is referred to the app functionality. Articles fall in this category if the app is actively used and some information must be provided to the app. An example of items in this category are the type of login used by the app (email or an external provider such as Facebook) and if user registration or password are needed to use the app. - **In-app information**: As in previous category, the app is analyzed from the inside to look for some information related to privacy such as, for example, information about security measures or information about data sharing. Analysis of privacy policies is assessed in a separate item, because some articles do that way. - **Personal information types:** To fall into this category, the article must make an explicit analysis of the type of personal data collected by the app. - **App communications**: Some articles analyze if the app communications are private by intercepting traffic. That way, it is possible to know not only if traffic is encrypted but also, in some cases, to check the content of the traffic. Some authors also find out the traffic destination of app’s communications, like third parties and ad sites. - **Static/dynamic analysis**: The use of static and/or dynamic analysis is very common when evaluating the security of an app. But it can also be used to analyze certain aspects of privacy such as, for example, find out if privacy in communications is properly implemented in the app or the types of permissions used by an app. - **Existence of a privacy policy**: the articles check the existence of a privacy policy. - Analysis of the **content of the privacy policy** and/or the ToS (Type of Service): the authors of the article have read the privacy policy and have searched for the presence or absence of certain information, such as how the data are stored, the use of encryption or if the data are shared with third parties, among others. Legibility (see next category) is excluded from this category because metrics used to evaluate legibility do not depend on the type of document being assessed. - **Privacy policy legibility:** Transparency is one of the pillars of GDPR. That way, some articles analyze some metrics of the apps regarding its readability. Metrics include the length of the document, the number of phrases and the use of readability algorithms available in literature. |
| **Basis of the assessment criteria (includes legal framework):**   - **Literature:** Authors used criteria defined by other authors in previous studies - **Authors’ experience:** Authors defined their own criteria based on their expertise - **Legal framework:** Authors defined criteria based on a legal framework such as the General Data Protection Regulations (GDPR) - **Privacy recommendations and principles:** Authors defined criteria based on privacy recommendations or principles |
| **Evaluation Criteria** |
| **Criteria:** (Select the items included in the evaluation criteria)   - Existence of a data controller - Data Protection Officer (DPO) details are given - Purposes of the processing are stated - Legal basis exists - Recipients of personal data are identified - International data transfers are disclosed - Period data are stored is stated - Existence of subject’s data rights - Existence of the right to withdraw consent - Existence of the right to complain a supervisory authority - Obligation to provide data - Existence of data processing/profiling - Nature of the collected information is disclosed - Risks of data collection are stated/managing confidentiality breaches - Location of the collected information is disclosed - User registration is required - Existence of a privacy policy - Privacy-policy good practices - Minimum data for the app functioning are collected - Protection minors/age of verification exists - Anonymization takes place |
| **Assessment of criteria:** Description of the assessment method used by authors. Examples of those assessments are:   - **Binary:** If the app complies the item (Yes or No) - **Tertiary:** Heuristics are valued as “Complies”, “Partially complies”, or “does not comply” - **Collection:** Points for meeting a group of items - **Formula:** Mathematical formula to calculate the privacy risk - **Percentage:** % of objects that meet the criteria - **Others:** For example, traffic monitoring |
| **Scoring Method** |
| **Score:** If authors included a privacy scoring method in their studies |
| **Weighted score:** If the scoring method includes weights for each assessed item |
