# Supplementary material for: Privacy Assessment in Mobile Health Apps: Scoping Review
Source: JMIR Mhealth Uhealth. 2020 Jul 2;8(7):e18868. doi: 10.2196/18868 (PMC7367524; doi:10.2196/18868)
Supplement: Multimedia Appendix 4 [file mhealth_v8i7e18868_app4.docx]

List of included studies

- M. Papageorgiou, E. Strigkos, E. Politou, A. Alepis, A. Solanas, and C. Patsakis, “Security and Privacy Analysis of Mobile Health Applications: The Alarming State of Practice,” IEEE Access, 2018.
- M. T. Minen, E. J. Stieglitz, R. Sciortino, and J. Torous, “Privacy Issues in Smartphone Applications: An Analysis of Headache/Migraine Applications,” Headache, 2018.
- K. Huckvale, J. Torous, and M. E. Larsen, “Assessment of the Data Sharing and Privacy Practices of Smartphone Apps for Depression and Smoking Cessation,” JAMA Netw. open, 2019.
- K. Scott, D. Richards, and R. Adhikari, “A review and comparative analysis of security risks and safety measures of mobile health apps,” Australasian Journal of Information Systems. 2015.
- T. Brüggemann, J. Hansen, T. Dehling, and A. Sunyaev. "An Information Privacy Risk Index for mHealth Apps. Proceedings of the Annual Privacy Forum (APF'16), pp. 190-201, 2016.
- A. Mense, S. Steger, M. Sulek, D. Jukic-Sunaric, and A. Mészáros. "Analyzing Privacy Risks of mHealth Applications." Studies in Health Technology and Informatics, vol. 221, pp. 41-5, 2016.
- L. Hutton et al., “Assessing the privacy of mhealth apps for self-tracking: Heuristic evaluation approach,” JMIR mHealth uHealth, 2018.
- B.C. Zapata, A. Hernández-Niñirola, J.L. Fernández-Alemán, and A. Toval. "Assessing the privacy policies in mobile personal health records". 36th Annual International Conference of the IEEE Engineering in Medicine and Biology Society (EMBC’14) , pp. 4956-4959, 2014.
- A. Sunyaev, T. Dehling, P. L. Taylor, and K. D. Mandl, “Availability and quality of mobile health app privacy policies,” J. Am. Med. Informatics Assoc., 2015.
- S. Leigh, J. Ouyang, and C. Mimnagh, “Effective? Engaging? secure? applying the orcha-24 framework to evaluate apps for chronic insomnia disorder,” Evid. Based. Ment. Health, 2017.
- A. Baumel, K. Faber, N. Mathur, J. M. Kane, and F. Muench. "Enlight: A Comprehensive Quality and Therapeutic Potential Evaluation Tool for Mobile and Web-Based eHealth Interventions", Journal of Medical Internet Research, 19 (3), e82, 2017. Baumel, 2017
- M. Bachiri, A. Idri, J.L. Fernández-Alemán, A. Toval. "Evaluating the Privacy Policies of Mobile Personal Health Records for Pregnancy Monitoring", Journal of Medical Systems, vol.42 (8), pp. 1-14, 2018.
- M.A. Robustillo-Cortés, M.R. Cantudo-Cuenca, R. Morillo-Verdugo, E. Calvo-Cidoncha. "High quantity but limited quality in healthcare applications intended for HIV-infected patients", Telemedicine and e-Health, 20 (8), pp. 729-735, 2014.
- A. Quevedo-Rodríguez and A.M. Wägner AM. "Mobile phone applications for diabetes management: a systematic review". Endocrinol Diabetes Nutr, vol. 66(5), pp.330-337, 2019.
- K. Knorr, D. Aspinall, and M. Wolters, “On the privacy, security and safety of blood pressure and diabetes apps,” in IFIP Advances in Information and Communication Technology, 2015.
- B.C. Zapata, A. Hernández-Niñirola, J.L. Fernández-Alemán, and A. Toval. "Privacy and Security in Mobile Personal Health Records for Android and iOS", RISTI - Revista Iberica de Sistemas e Tecnologias de Informação, vol. 13, pp. 35-50, 2014.
- P. Bondaronek, G. Alkhaldi, A. Slee, F. L. Hamilton, and E. Murray, “Quality of publicly available physical activity apps: Review and content analysis,” JMIR mHealth and uHealth. 2018.
- K. O’Loughlin, M. Neary, E. C. Adkins, and S. M. Schueller, “Reviewing the data security and privacy policies of mobile apps for depression,” Internet Interv., 2019.
- R. Adhikari, D. Richards, and K. Scott, “Security and privacy issues related to the use of mobile health apps,” in Proceedings of the 25th Australasian Conference on Information Systems, ACIS 2014, 2014.
- M. Aliasgari, M. Black, and N. Yadav. "Security Vulnerabilities in Mobile Health Applications", IEEE Conference on Applications, Information & Network Security (AINS2018), 2018.
- A. Mense, P. Urbauer, S. Sauermann, and H. Wahl. "Simulation environment for testing security and privacy of mobile health apps. Modeling and Simulation in Medicine Symposium (MSM ’16), Article 2, pp. 1–5, 2016.
- A. Powell, P. Singh, and J. Torous, “The Complexity of Mental Health App Privacy Policies: A Potential Barrier to Privacy”, JMIR Mhealth Uhealth 2018;6(7):e158, doi: 10.2196/mhealth.9871.
- K. Huckvale, J. T. Prieto, M. Tilney, P. J. Benghozi, and J. Car, “Unaddressed privacy risks in accredited health and wellness apps: A cross-sectional systematic assessment,” BMC Med., 2015
- J. M. Robillard, et al. “Availability, readability, and content of privacy policies and terms of agreements of mental health apps”, Internet Interventions, Volume 17, no. 100243, 2019.
