# Supplementary material for: Privacy Assessment in Mobile Health Apps: Scoping Review
Source: JMIR Mhealth Uhealth. 2020 Jul 2;8(7):e18868. doi: 10.2196/18868 (PMC7367524; doi:10.2196/18868)
Supplement: Multimedia Appendix 5 [file mhealth_v8i7e18868_app5.docx]

| **Reference / Object of the assessment** | App properties and behavior | In-app information | Personal information types | Static/dynamic analysis | App communications | Existence of a privacy policy | Content of the privacy policy | Privacy policy legibility |
| --- | --- | --- | --- | --- | --- | --- | --- | --- |
| Papageorgiou et al, 2018 |  | X |  | X | X | X | X |  |
| Minen et al, 2018 | X |  |  |  |  | X | X |  |
| Huckvale et al, 2019 |  | X |  |  | X | X | X |  |
| Scott et al, 2015 | X |  |  |  |  | X |  |  |
| Brüggemann et al, 2016 | X |  | X |  | X |  |  |  |
| Mense et al, 2016 |  |  |  |  | X |  |  |  |
| Hutton et al, 2018 | X | X |  |  |  | X | X |  |
| Zapata et al, 2014 | X |  |  |  |  | X |  |  |
| Sunyaev et al, 2015 |  |  |  |  |  | X | X | X |
| Leigh et al, 2017 |  | X |  |  |  | X | X |  |
| Baumel et al, 2017 |  |  |  |  |  | X | X |  |
| Bachiri et al, 2018 | X |  |  |  |  | X |  |  |
| de las Aguas Robustillo Cortés et al, 2014 | X | X |  |  |  |  |  |  |
| Quevedo-Rodríguez and Wagner, 2019 | X | X |  |  |  | X | X |  |
| Knorr et al, 2015 |  |  |  | X | X | X | X |  |
| Zapata et al, 2014 |  |  |  |  |  |  |  | X |
| Bondaronek et al, 2018 |  |  |  |  |  | X | X |  |
| O’Laughlin et al, 2019 |  |  |  |  |  | X | X |  |
| Adhikari et al, 2014 | X | X |  |  |  | X |  |  |
| Aliasgari et al, 2018 |  |  |  |  | X |  |  |  |
| Mense et al, 2016 |  |  |  |  | X |  |  |  |
| Powell el al, 2018 |  |  |  |  |  | X |  | X |
| Huckvale et al, 2015 | X | X | X |  | X | X | X |  |
| Robillard et al, 2019 |  |  |  |  |  | X | X | X |
