# Supplementary material for: Privacy Assessment in Mobile Health Apps: Scoping Review
Source: JMIR Mhealth Uhealth. 2020 Jul 2;8(7):e18868. doi: 10.2196/18868 (PMC7367524; doi:10.2196/18868)
Supplement: Multimedia Appendix 6 [file mhealth_v8i7e18868_app6.docx]

Data of selected studies

| **General Information** |
| --- |
| **Authors:** Papageorgiou et al. |
| **Title:** Security and Privacy Analysis of Mobile Health Applications: The Alarming State of Practice |
| **Year:** 2018 |
| **Source Title:** IEEE Access |
| **Source Type:** Journal |
| **App area:** 3 categories: (i) pregnancy and baby growth, (ii) personal/family members' health agenda and symptoms assistants/checkers, (iii) blood pressure and diabetes support. Curiously, they state they cannot disclose the names (or other identifiers) of the analyzed apps due to legal issues. |
| **Analyzed apps:** 20 |
| **Evaluation Procedure** |
| **Assessment Design:** Privacy and security evaluation |
| **Object of assessment:**   - Static/dynamic analysis - In-app information - App communications - Existence of privacy policy - Content of privacy policy |
| **Basis of the assessment criteria (includes legal framework):** Authors/GDPR |
| **Evaluation Criteria** |
| **Criteria:**  1. An updated study on the existence, relevance, quality and overall validity of the provided privacy policies. 2. Permission analysis (Android code analysis, identifying dangerous permissions); 3. Static code analysis (APK analysis, identifying some security aspects); 4. Data transmission on the internet (sending data to the owner; sending data via HTTP; sharing with third parties; number of third parties; number of third parties over HTTP); 5. Transmission of user location (same elements as in point 4); 6. Transmission of email and device ID; 7. HTTPS; 8 Use of chats (HTTPS); 9. SSL; 10. Reassessment of the privacy policy to inform the applications of the initial report; 11. Secure transmission of user data main problems; 12. Compliance with GDPR: Consent (I): Information about privacy policy before registration (11/19) Consent (II): Request consent before adding new information (1/19) Consent (III): Fill out an electronic questionnaire when participating (0/19) Right to withdraw consent (7/19) Right to data portability (7/19). Non-functional requirements: - DPO: None, but 12/19 give contact information; - Information on the collection and processing of information. (11/19) - Transfer to third countries (8/19) |
| **Assessment of criteria:** Apps that comply the items |
| **Scoring Method** |
| **Score:** No |
| **Weighted score:** No, though there are “major issues” and “minor issues” |

**Papageorgiou et al. (2018).**

| **General Information** |
| --- |
| **Authors:** Minen et al |
| **Title:** Privacy Issues in Smartphone Applications: An Analysis of Headache/Migraine Applications |
| **Year:** 2018 |
| **Source Title:** Headache |
| **Source Type:** Journal |
| **App area:** Headache |
| **Analyzed apps:** 14 |
| **Evaluation Procedure** |
| **Assessment Design:** Privacy evaluation |
| **Object de assessment:**   - App properties - Existence of privacy policy - Content of privacy policy |
| **Basis of the assessment criteria (includes legal framework):** Though HIPAA o FTC are mentioned, it is not clear how items are extracted. We consider that criteria are defined by the authors |
| **Evaluation Criteria** |
| **Criteria:**  1. Functionality for an online account. 2. Data Storage. 2.1. Stored locally on the user’s device. 2.2. Stored on remote servers. 2.3. Stored locally but provided optional Dropbox remote backups. 2.4. Stored locally but provided a paid option to store the diary data on the app provider’s servers. 2.5. Unclear whether stored locally or remotely. 3 Of those with a Privacy Policy: 3.1. Contained some description regarding the type of information collected from users- 3.2. Stated whether or not the app collects and stores any information (not only including diary data) remotely. 3.3. Stated whether or not the app could store diary entries on a remote server (or only locally). 3.4. Contained only vague statements about data collection. 3.5. Stated that data could be shared for medical research (including in anonymized format). 3.6. Permitted sharing of headache diary information directly with medical providers. 3.7. Stated that data could be shared for marketing, promotions, and/or advertising. 3.8. If there was an option to store data remotely (not including Dropbox only functionality), stated they only shared anonymized data with third parties in an aggregated manner unless users specifically requested disclosure, e.g., to health providers. 3.9. Policies (including those for apps without remote headache diary functionality) stated why they shared data with third parties. 3.10. Discussed the use of children: 3.10.1. Specifically, prohibited children under the age of 13. 3.10.2. Allowed children to use the application with a parent’s supervision. 3.10.3. Stated “we do not specifically market to children under the age of 13,” but did not explicitly prohibit children from using the application. 3.14. Explicitly granted a right of 3.14.1. Access to a user’s data. 3.14.2. Correct user’s data. 3.14.3. Delete user’s data. 3.14.4. Claims no personal data collected |
| **Assessment of criteria:** Apps that comply different items |
| **Scoring Method** |
| **Score:** No |
| **Weighted score:** N/A |

**Minen et al. (2018).**

| **General Information** |
| --- |
| **Authors:** Huckvale et al. |
| **Title:** Assessment of the Data Sharing Privacy Practices of Smartphone Apps for Depression and Smoking Cessation |
| **Year:** 2019 |
| **Source Title:** Jama Network open |
| **Source Type:** Journal |
| **App area:** Depression and smoking cessation |
| **Analyzed apps:** 36 apps (android and iOS) |
| **Evaluation Procedure** |
| **Assessment Design:** Privacy evaluation |
| **Object of the assessment:**   - App communications - In-app information - Existence of privacy policy - Content of privacy policy |
| **Basis of the assessment criteria (includes legal framework):** Privacy policy quality criteria from literature |
| **Evaluation Criteria** |
| **Criteria:**  Privacy criteria: Apps with a privacy policy, primary uses of data, secondary uses of data, send data to online services, send data to third parties, send data for analytics or research, send data while loading content, asserting nonidentifiable data collection only, technical and procedural security arrangements, how long data retained, inherent risks or limitations of security using public internet, how cookies will be used, procedures for opting-out online data sharing, consequences of not providing data, procedures for subject access requests, procedures for editing data held by developers or third parties, procedures for deleting data held by developers or third parties, complains procedures, special procedures for vulnerable or at-risk users and/or children, identity of the data controller, legal jurisdiction, legal jurisdiction governing data processing, date of policy, date next review, procedures for changing the terms of the policy, procedures after takeover or dissolution of legally responsible body. To see if app behaves as privacy policy says: MitM attack and tagging the destination (developer or third party) and data transmitted. |
| **Assessment of criteria:** Privacy criteria: % of privacy policies that meet the criteria. They monitor app’s traffic (MitM) and classify where the data is transmitted |
| **Scoring Method** |
| **Score:** No |
| **Weighted score:** N/A |

**Huckvale et al. (2019).**

| **General Information** |
| --- |
| **Authors:** Scott et al. |
| **Title:** A Review and Comparative Analysis of Security Risks and Safety Measures of Mobile Health Apps |
| **Year:** 2015 |
| **Source Title:** Australasian Journal of Information Systems |
| **Source Type:** Journal |
| **App area:** General health (most used apps) |
| **Analyzed apps:** 20 |
| **Evaluation Procedure** |
| **Assessment Design:** Privacy and security evaluation |
| **Object of the assessment:**   - App properties - Existence of privacy policy |
| **Basis of the assessment criteria (includes legal framework):** Identified from the literature |
| **Evaluation Criteria** |
| **Criteria:**  1. Does the app ask for user registration (name, address, birthday and email)? 2. Data stored in the cloud. 3.  Is consumer data shared with a third party or advertiser? 4. Does an app ask for user authentication (username and password)? 5. Are consumers able to update and correct their personal profiles? 6. Can consumers delete any personal information completely? 7. Data stored locally? 8. Are consumers informed about any data privacy and security measures? 9. Is there a privacy policy? |
| **Assessment of criteria:** 1-3: Risk score (1 point if there is a risk); 4-9. Safety score (1 point if it is safe). |
| **Scoring Method** |
| **Score:** Yes. Risk score: 0-3; Safety score: 0-6 |
| **Weighted score:** No |

**Scott et al. (2015).**

| **General Information** |
| --- |
| **Authors:** Brüggemann et al. |
| **Title:** An information privacy risk index for mHealth apps |
| **Year:** 2016 |
| **Source Title:** Proceedings of the Annual Privacy Forum |
| **Source Type:** Journal |
| **App area:** “Medical” and “Health and Fitness” |
| **Analyzed apps:** 298 (147 iOS; 151 Android) |
| **Evaluation Procedure** |
| **Assessment Design:** Privacy evaluation |
| **Object of the assessment:**   - App properties - Personal information types - App communications |
| **Basis of the assessment criteria (includes legal framework):** Apparently under the authors’ criteria, based on the literature |
| **Evaluation Criteria** |
| **Criteria:**  1. Information Sharing Targets (T); 2. Personal Information Types (13 types) (P); 3. Login (L); 4. Connection Security (S); 5. Unspecific Information Transfer (U); 6. Reasonable Information Collection (R) |
| **Assessment of the criteria:** PrivacyRiskScoreApp = TApp ∗ w(T) + PApp ∗ w(P) + LApp ∗ w(L)+ SApp ∗ w(S) + UApp ∗ w(U) + RApp ∗ w(R) |
| **Scoring Method** |
| **Score:** Yes. S, T, U and R are binary (L too?). P is the more elaborated: The authors identified 13 types of personal information input, but the maximum number of personal information input types identified for a single app was 5. This would lead to a single app never reaching the maximum score of 1.0. To remedy this, a correction factor is applied to the final privacy risk score. |
| **Weighted score:** Yes. It can be configured by the user |

**Brüggemann et al. (2016).**

| **General Information** |
| --- |
| **Authors:** Mense et al. |
| **Title:** Analyzing Privacy Risks of mHealth Applications |
| **Year:** 2016 |
| **Source Title:** Studies in Health Technology and Informatics |
| **Source Type:** Book chapter |
| **App area:** Health and fitness |
| **Analyzed apps:** 10 |
| **Evaluation Procedure** |
| **Assessment Design:** Privacy evaluation |
| **Object of the assessment:**   - App communications |
| **Basis of the assessment criteria (includes legal framework):** Authors |
| **Evaluation Criteria** |
| **Criteria:** 1. use of SSL; 2. use of certificate pinning; 3. type of information sent; 4. identify third parties; 5. identify if GPS, device-ID email address info is sent; |
| **Assessment of the criteria:** It is checked how many apps accomplish with the criteria |
| **Scoring Method** |
| **Score:** No |
| **Weighted score:** N/A |

**Mense et al. (2016).**

| **General Information** |
| --- |
| **Authors:** Hutton et al. |
| **Title:** Assessing the Privacy of mHealth Apps for Self-Tracking: Heuristic Evaluation Approach |
| **Year:** 2018 |
| **Source Title:** JMIR mHealth and uHealth |
| **Source Type:** Journal |
| **App area:** Self-tracking |
| **Analyzed apps:** 64 (Android) |
| **Evaluation Procedure** |
| **Assessment Design:** Privacy evaluation |
| **Object of the assessment:** Not clear. We assume:   - App properties - In-app information - Existence of privacy policy - Content of privacy policy |
| **Basis of the assessment criteria (includes legal framework):** FTC’s Fair Information Practices, GDPR, STRAP Framework, Literature |
| **Evaluation Criteria** |
| **Criteria:**  26 heuristics (4 categories): CAT. 1. Notice/awareness. H1. Before data are shared with a remote actor, the entity collecting the data is explicitly identified. H2. Before data are shared with a remote actor, the uses of the data are explicitly identified. H3. Before data are shared with a remote actor, the potential recipients are explicitly identified. H4. The nature and means of the data collected are explicitly identified. H5. Steps taken to ensure confidentiality, integrity, and quality of data are explained. H6. For those of above satisfied, notice is sufficiently explicit. H7. Can control when data are used for nonoperational secondary use, such as marketing or research. CAT. 2. Choice or Consent. H8. Consent acquired before data shared with remote actor. H9. Consent is explicitly opt-in: no preticked checkboxes, etc. H10. Can choose which data types are automatically collected from sensors or other sources, for example, connect a finance app to a single bank account or track steps but not heart rate. H11. Data collection consent is dynamic: if new types of data are being collected, consent is renewed in situ. H12. Data processing consent is dynamic: if the purpose of processing changes, consent is renewed. H13. Data distribution consent is dynamic: if the actors’ data are distributed to changes, consent is renewed. H14. Consent to store and process data can be revoked at any time: with the service and any other actors. H15. Can control where data are stored. CAT. 3. Access or Participation. H16. All raw collected data can be extracted from the service (in-app or via vendor’s website). H17. All data are available in standard text formats (CSVb, XML, JSONc, GPXd, etc). H18. Data extraction is available from within the service, for example, without raising a request with support. H19. Programmatic access to data is possible, for example, app programming interfaces are exposed. CAT. 4. Social Disclosure Usability. H20. Privacy controls are per-disclosure, for example, individual workouts can be published to a social networking site, not relying solely on global defaults. H21. Privacy controls allow granular sharing of data types, for example, when sharing a workout, the distance can be shared but not the pace. H22. Error prevention: is explicit confirmation acquired before a disclosure? H23. Minimize user memory load: Effects of a disclosure are visible throughout the disclosure flow (ie, memory of earlier decisions not required). H24. Minimalist: During the disclosure flow no extraneous information (such as adverts or irrelevant user interface elements) is displayed. H25. Consistency: Information shown during the disclosure flow is consistent with the effect of the disclosure. H26. Help and documentation: Contextual help with making privacy decisions is available |
| **Assessment of criteria:** Most of the heuristics are valued from 0-2 (0-1-2), though some of them have slightly different values (0/1, 0-3, or 0-4) |
| **Scoring Method** |
| **Score:** The paper does not show a score, but how different heuristics are accomplished. However, it is easy to assign a score to every app with the available information |
| **Weighted score:** N/A, but it could be (see Score above) |

**Hutton et al. (2018).**

| **General Information** |
| --- |
| **Authors:** Zapata et al. |
| **Title:** Assessing the privacy policies in mobile personal health records |
| **Year:** 2014 |
| **Source Title:** 2014 36th Annual International Conference of the IEEE Engineering in Medicine and Biology Society, EMBC 2014 |
| **Source Type:** Conference proceedings |
| **App area:** PHR apps |
| **Analyzed apps:** 24 (Android/iOS) |
| **Evaluation Procedure** |
| **Assessment Design:** Privacy evaluation |
| **Object of the assessment:**   - App properties - Existence of privacy policy |
| **Basis of the assessment criteria (includes legal framework):** The questionnaire criteria were extracted from the HIPAA Privacy Rule and based on the principles analyzed by a previous study that reviews the Privacy Policies of web PHRs |
| **Evaluation Criteria** |
| **Criteria:**  1.  Can the Privacy Policy be easily accessed? 2. Are changes to the Privacy Policy notified? 3. Does the mPHR include a strong authentication mechanism? 4. Are the data encrypted? 5. Does the mPHR follow any security standards or laws? 6. Does the mPHR allow multiple users, and if so, can access be granted and revoked? |
| **Assessment of criteria:** All 6 questions are valued as 0-0.5-1 |
| **Scoring Method** |
| **Score:** Yes |
| **Weighted score:** No |

**Zapata et al. (2014).**

| **General Information** |
| --- |
| **Authors:** Sunyaev et al. |
| **Title:** Availability and quality of mobile health app privacy policies |
| **Year:** 2015 |
| **Source Title:** Journal of the American Medical Informatics Association |
| **Source Type:** Journal |
| **App area:** Health and Fitness / Medical |
| **Analyzed apps:** 600 (Android/iOS) |
| **Evaluation Procedure** |
| **Assessment Design:** Privacy evaluation |
| **Object of the assessment:**   - Existence of privacy policy - Content of privacy policy - Privacy policy legibility |
| **Basis of the assessment criteria (includes legal framework):** Authors |
| **Evaluation Criteria** |
| **Criteria:**  1. Availability. 2. Characteristics (a. length, b. reading grade level (RGL) c. scope, d. transparency, - information shared with third parties, rationale for collection, sharing of information, user controls -. |
| **Assessment of criteria:** It is checked how many apps accomplish with the criteria |
| **Scoring Method** |
| **Score:** No |
| **Weighted score:** N/A |

**Sunyaev et al. (2015).**

| **General Information** |
| --- |
| **Authors:** Leigh et al |
| **Title:** Effective? Engaging? Secure? Applying the ORCHA-24 framework to evaluate apps for chronic insomnia disorder |
| **Year:** 2017 |
| **Source Title:** Evidence-Based Mental Health |
| **Source Type:** Journal |
| **App area:** Chronic insomnia |
| **Analyzed apps:** 19 (18 Android + 1 iOS) |
| **Evaluation Procedure** |
| **Assessment Design:** Multidimensional evaluation |
| **Object of the assessment:**   - In-app information - Existence of privacy policy - Content of privacy policy |
| **Basis of the assessment criteria (includes legal framework):** Data Protection Act 1998; Data Protection Directive; Information Commissioner’s Office; The Charter of Fundamental Rights of the EU; BSIgroup; GSMA; App quality alliance; authors; W3C. |
| **Evaluation Criteria** |
| **Criteria**  8 of 24 items deal with privacy: (1) Does the app state that no data will be shared with other parties without explicit user consent? (2) Does the app outline a process for managing data confidentiality breaches? (3) Is there a data privacy policy, either within the app itself or on a website? (4) Does the data privacy policy, or statement, provide detail about what data is collected by the app? (5) Does the data privacy policy, or statement, provide detail about what that data is used for by the app? (6) Does the data privacy policy, or statement, state whether personal data are stored using recognized secure data storage technologies? (7) Does the data privacy policy, or statement, state that all personally identifiable data will be encrypted in transit between the device and any developer host storage? (e.g., using FTP protocol) (8) Does the data privacy policy state that only the minimum data items necessary for the app to function will be collected? |
| **Assessment of criteria:** App privacy features (1-2), Privacy policy (3-8) |
| **Scoring Method** |
| **Score:** Yes, 1 point per question, 0-8 |
| **Weighted score:** No |

**Leigh et al. (2017).**

| **General Information** |
| --- |
| **Authors:** Baumel et al. |
| **Title:** Enlight: A comprehensive quality and therapeutic potential evaluation tool for mobile and web-based eHealth interventions |
| **Year:** 2017 |
| **Source Title:** Journal of Medical Internet Research |
| **Source Type:** Journal |
| **App area:** Health Related Behaviors; Mental Health |
| **Analyzed apps:** 84 (includes 42 web apps and 42 mobile apps) |
| **Evaluation Procedure** |
| **Assessment Design:** Multidimensional evaluation |
| **Object of the assessment:**   - In-app information - Existence of privacy policy - Content of privacy policy |
| **Basis of the assessment criteria (includes legal framework):** a comprehensive systematic review was performed to identify relevant quality rating criteria in line with the PRISMA statement |
| **Evaluation Criteria** |
| **Criteria:**  1. The system informs users of the data journey in detail, so they understand all sources of data exposure (and risks if their device or app are not password protected). This includes data stored on servers and on the device 2. The system notifies users how their personal identifiable information will be kept confidential and secured. 3. The system notifies users about how gathered data may be used (e.g., for commercial reasons). 4. For programs explicitly designed to be used by minors, the system includes a section requiring the approval/supervision of a legal guardian. 5. The system explicitly tunnels users through the terms of use (privacy/data wise, including items #1-#3, and #4 [if applicable]) before program utilization. In cases in which all other items are N/A, the system generally states that it does not collect any data, identifications, etc. 6. The system enables users to keep identifiers private (and this is the default setting). 7. It is apparent when information will be seen by other users/members even if data do not contain identifiers (e.g., when they are in a particular zone where data are not kept private). 8. The system warns users about providing private identifiable information (e.g., name, health information, home address) to other users on the platform. |
| **Assessment of criteria:** Terms of use (1-5), Systems with in-House Social Platforms. 0-8 (1 point if the app does not follow the criteria). 0 points is maximum privacy |
| **Scoring Method** |
| **Score:** Yes. 0-8 (1 point if the app does not follow the criteria). 0 points is maximum privacy |
| **Weighted score:** No |

**Baumel et al. (2017).**

| **General Information** |
| --- |
| **Authors:** Bachiri et al. |
| **Title:** Evaluating the Privacy Policies of Mobile Personal Health Records for Pregnancy Monitoring |
| **Year:** 2018 |
| **Source Title:** Journal of Medical Systems |
| **Source Type:** Journal |
| **App area:** Pregnancy |
| **Analyzed apps:** 19 (iOS and Android) |
| **Evaluation Procedure** |
| **Assessment Design:** Privacy evaluation |
| **Object of the assessment:**   - App properties - Existence of privacy policy |
| **Basis of the assessment criteria (includes legal framework):** Based on Literature (own work) PHR for web adapted by authors. |
| **Evaluation Criteria** |
| **Criteria:**  35 items into 10 categories: 1. Privacy Policy Location (PPL): PPL1: Accessible via the mPHR; 2. Notification of changes to privacy policy (CPP): CPP1: Change notification; CPP2: Change notification directly; 3. Access Management (AM): AM1: Users grant access; AM2: Users grant access to healthcare professionals; AM3: Users grant access to people with other roles; AM4: Kinds of permissions; AM5: Access in case of emergency; 4. Security Data Management (DM): DM1: User adds, modifies, removes and updates information; DM2: Healthcare professionals update or add information; DM3: Family members’ data; DM4: Connection with other PHRs / EHRs; DM5: Monitoring devices; 5. Data accessed without the user’s permission (DA): DA1: Not accessed or information related to the users’ accesses; 6. Access Audit (AA): AA1: Who has accessed it; AA2: With what aim; 7. Access Criteria (AC): AC1: Roles; AC2: Groups; AC3: Location; AC4: Time; AC5: Transaction; 8. Type Authentication (AU): AU1: Something known; AU2: Something the user has; AU3: Biometric factors; 9. Safeguards (S): S1: Physical security measures; S2: Limited access; S3: Electronic security measures; S4: Encrypted data; S5: Backup system; S6: Defined data security plan; S7: Staff training; S8: Privacy seal; 10. Standards and Regulations (ST): ST1: HIPAA considered; ST2: HIPAA; ST3: Health on the Net Foundation Code of Conduct (HONcode) characteristic. |
| **Assessment of criteria:** Number of complied criteria. |
| **Scoring Method** |
| **Score:** Yes |
| **Weighted score:** No |

**Bachiri et al. (2018).**

| **General Information** |
| --- |
| **Authors:** Robustillo-Cortés et al. |
| **Title:** High quantity but limited quality in healthcare applications intended for HIV-infected patients |
| **Year:** 2014 |
| **Source Title:** Telemedicine and e-Health |
| **Source Type:** Journal |
| **App area:** VIH |
| **Analyzed apps:** 41 (iOS and Android) |
| **Evaluation Procedure** |
| **Assessment Design:** Multidimensional evaluation |
| **Object of the assessment:**   - App properties - In-app information |
| **Basis of the assessment criteria (includes legal framework):** Recommendations of the Happtique HACP (Health App Certification Programme), the FDA (Food and Drug Administration) Mobile Medical Applications, and recommendations for health apps of ACSA (Agencia de Calidad Sanitaria de Andalucía). |
| **Evaluation Criteria** |
| **Criteria:**  Criteria related with privacy or security: 1. Does the app use a secure system for the transmission of sensitive data or personal data in e-commerce; 2. Before the app is downloaded, is it specified whether registration is required and the data collected for the same?; 3. Does it describe in the app, before discharge, the purpose and use of personal information?; 4. Does the app, prior to discharge, declare to whom personal information may be disclosed?; 5. Does the app state that third-party access is available following safety standards that guarantee confidentiality at all times?; 6. If the app provides access to the user accounts or social networking profiles, does it inform the user?; 7. Is there an age verification process for the user?; 8. Does the app report the security mechanisms used to protect information from unauthorized access?; 9. Does the app features a locking mechanism for access to personal information by PIN, password, or other protection system?; 10. Does the app have a reliable password reminder?; 11. Does the app declare the nature, terms, and conditions of services in the cloud?; 12. Can the information stored in the cloud be managed by users? |
| **Assessment of criteria:** –1, does not comply with item; 0, not applicable; or 1, complies the item. |
| **Scoring Method** |
| **Score:** Yes, though it is a general app score |
| **Weighted score:** Yes (weighted by experts) |

**Robustillo-Cortés et al. (2014).**

| **General Information** |
| --- |
| **Authors:** Quevedo-Rodríguez and Wagner |
| **Title:** Mobile phone applications for diabetes management: A systematic review |
| **Year:** 2019 |
| **Source Title:** Endocrinología, Diabetes y Nutrición |
| **Source Type:** Journal |
| **App area:** Diabetes |
| **Analyzed apps:** 42 (free, in Spanish) |
| **Evaluation Procedure** |
| **Assessment Design:** Multidimensional evaluation |
| **Object of the assessment:**   - App properties - In-app information - Content of privacy policy |
| **Basis of the assessment criteria (includes legal framework):** Recommendations of SAS (Andalusian Health Service), AppSaludable certification. |
| **Evaluation Criteria** |
| **Criteria:**  Inside privacy items, there are the following: 1. The app informs the user about the nature of the collected data and the purpose of this collection, about access policies and data processing, and about commercial agreements with third parties. 2. The application describes the terms and conditions on the stored data. 3. The application preserves the privacy of the information, contains express user consents and warns of the risks of using the mobile app. 4. Security measures are ensured when data must be collected or exchanged. 5. The app ensures the access to stored information and informs about changes in its privacy policy; 6. There are measures for the protection of minors. 7. The app informs the user about when accessing to other resources inside the device. |
| **Assessment of criteria:** Compliance of the items: 2 complies, 1 partially complies, 0 does not comply |
| **Scoring Method** |
| **Score:** Yes, but inside the global app quality. |
| **Weighted score:** No |

**Quevedo-Rodríguez and Wagner (2019).**

| **General Information** |
| --- |
| **Authors:** Knorr et al. |
| **Title:** On the privacy, security and safety of blood pressure and diabetes apps |
| **Year:** 2015 |
| **Source Title:** IFIP Advances in Information and Communication Technology |
| **Source Type:** Journal |
| **App area:** Diabetes and blood pressure. |
| **Analyzed apps:** 154 |
| **Evaluation Procedure** |
| **Assessment Design:** Privacy and security evaluation |
| **Object of assessment:**   - Static/dynamic analysis - App communications - Existence of privacy policy - Content of privacy policy |
| **Basis of the assessment criteria (includes legal framework):** Privacy principles of the OECD |
| **Evaluation Criteria** |
| **Criteria:**  1. Existence of privacy policy; 2. Length of privacy policies; 3. Date of the policy; 4. Responsible; 5. Security safeguards; 6. Openness; 7. Purpose specified; 8. Individual participation; 9. Data for other purposes; 10. Storage in third parties; 11. Sale of data; 12. Sharing with third parties.  In the static analysis, the use of SSL is verified (MitM vulnerability or weak cypher). Traffic is analiyzed and app permissions are checked. |
| **Assessment of criteria:** General compliance of items. |
| **Scoring Method** |
| **Score:** No |
| **Weighted score:** N/A |

**Knorr et al. (2015).**

| **General Information** |
| --- |
| **Authors:** Zapata et al. |
| **Title:** Privacy and Security in Mobile Personal Health Records for Android and iOS |
| **Year:** 2014 |
| **Source Title:** RISTI - Revista Ibérica de Sistemas e Tecnologias de Informação |
| **Source Type:** Journal |
| **App area:** PHR apps |
| **Analyzed apps:** 24 mPHR |
| **Evaluation Procedure** |
| **Assessment Design:** Privacy evaluation |
| **Object of assessment:**   - Content of privacy policies |
| **Basis of the assessment criteria (includes legal framework):** Evaluation based on questionnaire conducted by two of the authors. The structuring of the questionnaire is based on the Principles of Good Information Practices (FIPPs) of the Federal Trade Commission (FTC). |
| **Evaluation Criteria** |
| **Criteria:**  NOTIFICATION. C1 Is the privacy policy easily accessible? C2 Are changes in the privacy policy notified to the user? C3 Is the user informed of the use of Cookies, data analysis services, geolocation services or IP storage? C4 Does the mPHR follow any safety standards or recommendations? ELECTION C5 Is there any mechanism to access data in case of medical emergency? C6 If the mPHR allows connection to other PHRs or EHRs, are the conditions explained? ACCESS C7 If the mPHR allows multiple users, can other users be granted and revoked? What kind of access does it allow? SECURITY C8 Does the mPHR use a strong authentication mechanism? C9 If data is stored locally, are they encrypted? What encryption mechanism is used? C10 If data is transferred online, are they encrypted? What encryption mechanism is used? What secure communication protocol is used? C11 If data is stored in the cloud, are they encrypted? What encryption mechanism is used? Is it explicit if there is access control, audit and physical protection of the servers? C12 If the mPHR allows the creation of backup copies, how is security guaranteed on that copy? |
| **Assessment of criteria:** 2 Complies item; 1 Partially complies item; 0 Does not comply item. |
| **Scoring Method** |
| **Score:** Yes |
| **Weighted score:** No |

**Zapata et al. (2014).**

| **General Information** |
| --- |
| **Authors:** Bondaronek et al. |
| **Title:** Quality of publicly available physical activity apps: Review and content analysis |
| **Year:** 2018 |
| **Source Title:** JMIR mHealth and uHealth |
| **Source Type:** Journal |
| **App area:** Physical activity |
| **Analyzed apps:** 65 |
| **Evaluation Procedure** |
| **Assessment Design:** Privacy and security evaluation |
| **Object of assessment:**   - Existence of privacy policy - Content of privacy policy |
| **Basis of the assessment criteria (includes legal framework):** based on the recommendations of the Information Commissioners Office and Online Trust Alliance |
| **Evaluation Criteria** |
| **Criteria:**  Q1: Availability. Is there privacy information available? (only continue if answered ‘Yes’) Q2: Availability Is the privacy information available without the need to download the app? Q3: Availability Is the privacy information available within the app? Q4: Accessibility Is there a short form notice (in plain English) highlighting key data practices which are disclosed in detail in the full privacy policy? Q5: Accessibility Is the privacy policy available in any other languages? Q6: Data gathering Does the app collect Personally Identifiable Information? Q7: Data sharing Does the app share users’ data with 3rd party? Q8: Data security Does the app say how the users' data security is ensured? e.g. encryption, authentication, firewall system |
| **Assessment of criteria:** Apps that comply different items |
| **Scoring Method** |
| **Score:** No, at least in the privacy items |
| **Weighted score:** N/A |

**Bondaronek et al. (2018).**

| **General Information** |
| --- |
| **Authors:** O'Laughlin et al |
| **Title:** Reviewing the data security and privacy policies of mobile apps for depression. |
| **Year:** 2019 |
| **Source Title:** Internet interventions |
| **Source Type:** Journal |
| **App area:** Depression |
| **Analyzed apps:** 116 |
| **Evaluation Procedure** |
| **Assessment Design:** Privacy evaluation |
| **Object of assessment:**   - Existence of privacy policy - Content of privacy policy |
| **Basis of the assessment criteria (includes legal framework):** Authors |
| **Evaluation Criteria** |
| **Criteria**  1. Does the app have a privacy policy? 2. Is there a login process to view and add data? 3. Does the privacy policy state that the data is encrypted or that the info is stored locally? 4. Are the information storage and sharing processes described? 5. Is it described if users can delete the info? 6. Is it described if users can edit the info? 7. Is it indicated if users can use the app without identifying information? |
| **Assessment of criteria: S**ome of the items received a white/light grey/dark grey score; others, a white/light grey score; one, a white/light grey/black score |
| **Scoring Method** |
| **Score:** Yes (Acceptable, inacceptable, questionable) |
| **Weighted score:** No |

**O’Laughlin et al. (2019).**

| **General Information** |
| --- |
| **Authors:** Adhikari et al. |
| **Title:** Security and privacy issues related to the use of mobile health apps |
| **Year:** 2014 |
| **Source Title:** Proceedings of the 25th Australasian Conference on Information Systems, ACIS 2014 |
| **Source Type:** Conference proceedings |
| **App area:** mHealth |
| **Analyzed apps:** Top 20 mHealth apps |
| **Evaluation Procedure** |
| **Assessment Design:** Privacy and security evaluation |
| **Object of assessment:**   - App properties - In-app information - Existence of privacy policy |
| **Basis of the assessment criteria (includes legal framework):** Identified from the literature |
| **Evaluation Criteria** |
| **Criteria:**  1. Does the app ask for user registration (name, address, birthday and email)? 2. Are consumers able to update and correct their personal profiles? 3. Does an app ask for user authentication (username and password)? 4. Can consumers delete any personal information completely? 5.Where is data stored (locally on a device or in a cloud)? 6. Is consumer data shared with a third party or advertiser? 7. Are consumers informed about any data privacy and security measures? 8. Is there a privacy policy? |
| **Assessment of the criteria:** Risk score (0-3, first 3 questions); Safe score (0-5, last 5) |
| **Scoring Method** |
| **Score:** Yes, risk score and safe score |
| **Weighted score:** No, but there is a distinction between scores |

**Adhikari et al. (2014).**

| **General Information** |
| --- |
| **Authors:** Aliasgari et al |
| **Title:** Security Vulnerabilities in Mobile Health Applications |
| **Year:** 2018 |
| **Source Title:** IEEE Conference on Application, Information and Network Security (AINS) |
| **Source Type:** Conference proceedings |
| **App area:** mHealth |
| **Analyzed apps:** Top 25 Android mHealth apps |
| **Evaluation Procedure** |
| **Assessment Design:** Privacy and security evaluation |
| **Object of assessment:**   - App communications |
| **Basis of the assessment criteria (includes legal framework):** Healthcare Insurance Portability and Accountability Act (HIPAA) |
| **Evaluation Criteria** |
| **Criteria:**  1. Use of a correct SSL configuration (TLS server type, key types, encryption methods, HSTS; 2. Sending unprotected data to servers (intercepted with MiTM); 3. Sensitive data collected with the MiTM (passwd, names, e-mails ...) 4. Compliance with HIPAA in its terms and conditions |
| **Assessment of criteria:** HIPAA Compliance or not. The authors checked if terms and conditions stated HIPAA compliance or asked the app’s support teams. |
| **Scoring Method** |
| **Score**: Yes, although there is no global score, there are certain scores on TLS and on HIPAA compliance |
| **Weighted score:** No |

**Aliasgari et al. (2018).**

| **General Information** |
| --- |
| **Authors:** Mense et al. |
| **Title:** Simulation environment for testing security and privacy of mobile health apps |
| **Year:** 2016 |
| **Source Title:** Proceedings of the Modeling and Simulation in Medicine Symposium |
| **Source Type:** Conference Proceedings |
| **App area:** Health and fitness |
| **Analyzed apps:** 10 |
| **Evaluation Procedure** |
| **Assessment Design:** Privacy and security evaluation |
| **Object of assessment:**   - App communications |
| **Basis of the assessment criteria (includes legal framework):** OWASP/Authors |
| **Evaluation Criteria** |
| **Criteria:**  1. Use of encrypted communication; 2. Possibility of intercepting the transfer of encrypted data; 3. Data transmission to third parties |
| **Assessment of criteria:** Compliance or not |
| **Scoring Method** |
| **Score:** No |
| **Weighted score:** N/A |

**Mense et al. (2016).**

| **General Information** |
| --- |
| **Authors:** Powell et al. |
| **Title:** The complexity of mental health app privacy policies: A potential barrier to privacy |
| **Year:** 2018 |
| **Source Title:** JMIR mHealth and uHealth |
| **Source Type:** Journal |
| **App area:** Diabetes vs. Mental health |
| **Analyzed apps:** 70 (41 diabetes + 29 mental health) |
| **Evaluation Procedure** |
| **Assessment Design:** Privacy evaluation |
| **Object of assessment:**   - Existence of privacy policy - Privacy policies legibility. |
| **Basis of the assessment criteria (includes legal framework):** Not clear. We consider that criteria are defined by the authors |
| **Evaluation Criteria** |
| **Criteria:**  word count, sentences per paragraph, words per sentence, characters per word, average number of sentences per 100 words, average words with six or more characters, average number of sentences per 100 words, Flesch Reading Ease, Flesch-Kincaid Grade Level, Gunning Fog Score, SMOG Index, Coleman Liau Index, Automated Readability Index, Fry Grade Level, and Raygor Estimate Graph Grade Level |
| **Assessment of criteria:** Average score / Median / Range for every item comparing diabetes apps vs mental health apps |
| **Scoring Method** |
| **Score:** Average score / Median / Range for every item |
| **Weighted score:** No |

**Powell et al. (2018).**

| **General Information** |
| --- |
| **Authors:** Huckvale et al. |
| **Title:**  Unaddressed privacy risks in accredited health and wellness apps: A cross-sectional systematic assessment |
| **Year:** 2015 |
| **Source Title:** BMC Medicine |
| **Source Type:** Journal |
| **App area:** Health apps |
| **Analyzed apps:** 79 |
| **Evaluation Procedure** |
| **Assessment Design:** Privacy and security evaluation |
| **Object of assessment:**   - App properties - Personal information types - In-app information - App communications - Existence of privacy policy - Content of privacy policy |
| **Basis of the assessment criteria (includes legal framework):** Data Protection Act |
| **Evaluation Criteria** |
| **Criteria:**  A. Privacy policies: 1. Privacy disclosure available; 2. In-app privacy policy; 3. Other privacy policy; 4. Policy mentions app; 5. Advertising policy; 6. No privacy disclosure; 7. In-app clinical disclaimer. B. Concordance of policies and data handling practices. C. Coverage of privacy and security-related topics in privacy policies. 1. Uses of data: Primary uses of collected data; Secondary uses of collected data; Sending data to developer-provided online services; Sending data to advertisers/marketers; Sending data for analytics/research; Sending data while loading content; Anonymous uses only. 2. Technical concerns: Technical and procedural security arrangements; How long data will be retained; Inherent risks or limitations of security on mobile device/internet; The use of cookies. 3. User rights: Procedures for opting out of data sharing; Consequences of not providing or sharing data; Procedures for subject access requests; Procedures for editing data held by developers/third parties; Procedures for deleting data held by developers/third parties; Complaints procedures; Special procedures for handling data for vulnerable users. 4. Administrative details: Identify data controller or responsible legal entity; Legal jurisdiction governing policy; Jurisdictions under which data will be processed; Date of policy; Date of next review; Procedures for changing the terms of the policy. |
| **Assessment of criteria:** Percentage of apps complying the different items |
| **Scoring Method** |
| **Score:** No |
| **Weighted score:** N/A |

**Huckvale et al. (2015).**

| **General Information** |
| --- |
| **Authors:** Robillard et al. |
| **Title:** Availability, readability, and content of privacy policies and terms of agreements of mental health apps |
| **Year:** 2019 |
| **Source Title:** Internet Interventions |
| **Source Type:** Journal |
| **App area:** Mental health apps |
| **Analyzed apps:** 369 apps (Android and iOS) |
| **Evaluation Procedure** |
| **Assessment Design:** Privacy evaluation |
| **Object of the assessment:**   - Existence of privacy policy - Content of privacy policy - Privacy policy legibility |
| **Basis of the assessment criteria (includes legal framework):** Readability: Readability calculator, average of Gunning Fog, Flesh Kincaid and SMOG (found in literature). Content: Own design. |
| **Evaluation Criteria** |
| **Criteria:**  Nature of the information collected (doesn’t describe, personal, aggregated, anonymous, personally identifiable, non-personal); types of information collected (contact, email, name, usage statistics, device info, location, phone, login info, gender, address, registration info, communications to, demographic info, age, date of birth, financial/billing, mood/emotions, health info, social media, photos, optional social media, comments, posts, messages, mobile carrier, thoughts/journal, diagnostic info). Uses of information (contacting user, improving the app, provide services, analysis/monitoring, personalize experience, administration, personalize advertisement, technical, billing, display info back to user). Nature of information shared with third parties (aggregated, anonymous, non-identifiable, personal, personally identifiable). Types of third parties (service providers, analytics service, partners, advertisers/vendors, researchers). Reasons for disclosing information (comply with legal process, sale of company/acquisition, merger, protect rights of developers, sale of assets, protect safety of others, protect property of developer, protect property of others, protect rights of others, protect safety of developer, prevent illegal activity, bankruptcy, investigate illegal activity, enforce agreement, violation of terms). License granted to developer (worldwide, non-exclusive, royalty-free, sub-licensable, perpetual, transferable, fully paid, unlimited). Licence privileges granted to developer (use, distribute, display, copy, modify, reproduce, create derivative works, publish, perform, transmit, adapt, translate, process, store, edit, excerpt, incorporate in other works. Also (scattered thru text but not appearing in figures): Consent implied, don’t sell personal or personal identifiable data, sell aggregated information, user info may be shared, user info won’t be shared unless consent, mentions security measures, developer cant warranty security, opt-out possible, can delete information, may delete information but with caveats, governing laws. |
| **Assessment of criteria:** The authors read privacy policies and ToA to see if each item referred before is present or not in them. Results are given as % of apps that meet the criteria |
| **Scoring Method** |
| **Score:** No |
| **Weighted score:** N/A |

**Robillard et al. (2019).**
